# Human-to-Bovine Jump of *Staphylococcus aureus* CC8 Is Associated with the Loss of a β-Hemolysin Converting Prophage and the Acquisition of a New Staphylococcal Cassette Chromosome

**DOI:** 10.1371/journal.pone.0058187

**Published:** 2013-03-11

**Authors:** Grégory Resch, Patrice François, Delphine Morisset, Milos Stojanov, Eve J. Bonetti, Jacques Schrenzel, Olga Sakwinska, Philippe Moreillon

**Affiliations:** 1 Department of Fundamental Microbiology, University of Lausanne, Lausanne, Switzerland; 2 Genomic Research Laboratory, University Hospital of Geneva, Geneva, Switzerland; 3 Laboratory of Bacteriology, University Hospital of Geneva, Geneva, Switzerland; 4 RUWAG Handels AG, Bettlach, Switzerland; 5 Nestlé Research Center, Lausanne, Switzerland; University of Edinburgh, United Kingdom

## Abstract

*Staphylococcus aureus* can colonize and infect both humans and animals, but isolates from both hosts tend to belong to different lineages. Our recent finding of bovine-adapted *S. aureus* showing close genetic relationship to the human *S. aureus* clonal complex 8 (CC8) allowed us to examine the genetic basis of host adaptation in this particular CC. Using total chromosome microarrays, we compared the genetic makeup of 14 CC8 isolates obtained from cows suffering subclinical mastitis, with nine CC8 isolates from colonized or infected human patients, and nine *S. aureus* isolates belonging to typical bovine CCs. CC8 isolates were found to segregate in a unique group, different from the typical bovine CCs. Within this CC8 group, human and bovine isolates further segregated into three subgroups, among which two contained a mix of human and bovine isolates, and one contained only bovine isolates. This distribution into specific clusters and subclusters reflected major differences in the *S. aureus* content of mobile genetic elements (MGEs). Indeed, while the mixed human-bovine clusters carried commonly human-associated β-hemolysin converting prophages, the bovine-only isolates were devoid of such prophages but harbored an additional new non-*mec* staphylococcal cassette chromosome (SCC) unique to bovine CC8 isolates. This composite cassette carried a gene coding for a new LPXTG-surface protein sharing homologies with a protein found in the environmental bacterium *Geobacillus thermoglucosidans*. Thus, in contrast to human CC8 isolates, the bovine-only CC8 group was associated with the combined loss of β-hemolysin converting prophages and gain of a new SCC probably acquired in the animal environment. Remaining questions are whether the new LPXTG-protein plays a role in bovine colonization or infection, and whether the new SCC could further acquire antibiotic-resistance genes and carry them back to human.

## Introduction

*Staphylococcus aureus* are major human and animal pathogens that can produce a variety of diseases, from relatively mild skin and soft tissue infections to life-threatening blood stream bacteremia and endocarditis [1], [2]. In addition, this bacterium is mastermind in developing antibiotic resistances, and some strains have become resistant to virtually all non-experimental drugs, including the whole family of β-lactam molecules in the case of methicillin-resistant *S. aureus* (MRSA) [3], as well as last-resort vancomycin and daptomycin [4], [5]. In humans, the major reservoir of *S. aureus* is represented by healthy carriers, who account for up to 30% of the population, and harbor the organism in their anterior nares and sometimes other anatomic sites [6]. Besides, *S. aureus* carriage was also reported in numerous animal species including dog, cat, horse, pig, poultry and cattle [7], [8], [9]. However, while *S. aureus* are quite ubiquitous in terms of host species, different animals tend to harbor different lineages (i.e. clonal complexes, or CCs for short) as recognized in pioneer work by Devriese and Oeding [10], and amply confirmed thereafter [11], [12], [13], [14], [15], [16], [17]. Several studies suggested that critical modulators of this host specificity might be mobile genetic elements (MGEs), gene decay, or adaptive evolution of surface proteins [11], [12], [14], [15], [18], [19], [20]. For instance, it has been suggested that the presence of the immune evasion cluster (IEC), a gene cluster carried by β-hemolysin converting bacteriophages, was strongly correlated with human isolates [21]. Such host-specific genes were suggested to be useful as epidemiologic markers [20].

We recently observed a close genetic relationship between *S. aureus* strains isolated from bovine suffering subclinical mastitis and strains of the prominent human CC8, suggesting recent human to bovine jump [17]. Here, we further compared the genetic makeup of human and bovine CC8 *S. aureus* strains, using a collection of epidemiologically independent isolates collected in Switzerland [17]. We observed evidence for a human to bovine jump rather than the contrary. Notably, the jump was associated with the loss of a β-hemolysin converting prophage typical of human strains [15], [22], [23], plus the acquisition of a new bovine-specific SCC element, which lacked the methicillin-resistance *mecA* gene, but carried a new LPXTG protein.

## Materials and Methods

### *S. aureus* Strains Selection

Nine epidemiologically unrelated human CC8 strains and 14 epidemiologically independent CC8 strains recovered from bovine subclinical mastitis (labeled “M”) were included in the study (Table 1). All strains were isolated from humans or animals in Western Switzerland. Concerning the human CC8 strains, three were recovered from healthy carriers and were labeled “Laus”, four were isolated from patients with bloodstream infections and were labeled “I”, and two corresponded to the reference strains USA300_FPR3757 (USA300) [24] and COL [25]. The bovine CC8 strains were chosen to represent all *spa* types found among 400 isolates previously collected [17], [26]. In addition, nine isolates from four typical bovine lineages (CC20, CC97, CC151, and CC479) were included.

**Table 1:** Genotyping results for cow and human *S. aureus* strains used in this study (according to ref. [11], [17], [44], and [45] for RF122, “M” strains, USA300, and COL, respectively).

CC/AFLP	Name of isolates	Source	Spa-type	Spa repeats	Sequence Type
8	COL*	Human infection	t008	11-19-12-21-17-34-24-34-22-25	250
	USA300*	Human wrist abscess	t008	11-19-12-21-17-34-24-34-22-25	8
	I2*	Human bloodstream infection	t008	11-19-12-21-17-34-24-34-22-25	8
	I29	Human bloodstream infection	t121	11-19-21-17-34-24-34-22-25	8
	I36	Human bloodstream infection	t008	11-19-12-21-17-34-24-34-22-25	8
	I37	Human bloodstream infection	t622	11-19-12-21-17-34-22-25	8
	Laus102	Human carriage	t008	11-19-12-21-17-34-24-34-22-25	8
	Laus270	Human carriage	t121	11-19-34-24-34-22-25	8
	Laus385	Human carriage	t2293	11-19-34-24-34-22-25	8
	M5	Bovine subclinical mastitis^1^	t2953	11-12-21-17-34-24-34-22-25-25	8
	M20	Bovine subclinical mastitis^1^	t5694	11-12-17-34-24-34-22-25-25	8
	M37	Bovine subclinical mastitis^1^	t024	11-12-21-17-34-24-34-22-25	8
	M86	Bovine subclinical mastitis^1^	t5271	11-17-34-24-34-22-25-25	8
	M117	Bovine subclinical mastitis^1^	t5694	11-12-17-34-24-34-22-25-25	8
	M124	Bovine subclinical mastitis^1^	t6281	04-21-17-34-24-34-22-25-25-75	8
	M160	Bovine subclinical mastitis^1^	t5270	11-12-21-17-34-24-34-22-25-25-25	8
	M186	Bovine subclinical mastitis^1^	t024	11-12-21-17-34-24-34-22-25	8
	M192	Bovine subclinical mastitis^1^	t5270	11-12-21-17-34-24-34-22-25-25-25	8
	M222	Bovine subclinical mastitis^1^	t5268	11-21-17-37-24-34-22-25-25	8
	M283	Bovine subclinical mastitis^1^	t2953	11-12-21-17-34-24-34-22-25-25	8
	M308	Bovine subclinical mastitis^1^	t711	04-21-17-34-24-34-22-25	8
	M313	Bovine subclinical mastitis^1^	t5268	11-21-17-34-24-34-22-25-25	8
	M319	Bovine subclinical mastitis^1^	t5271	11-17-34-24-34-22-25-25	8
20	M3	Bovine subclinical mastitis^1^	t164	07-06-17-21-34-34-22-34	389
	M159	Bovine subclinical mastitis^1^	t2094	26-06-17-21-34-34-22-34	389
	M323	Bovine subclinical mastitis^1^	t164	07-06-17-21-34-34-22-34	389
97	M32	Bovine subclinical mastitis^1^	t524	04–17	71
	M356	Bovine subclinical mastitis^1^	t524	04–17	71
151	RF122	Bovine subclinical mastitis^1^	t529	04–34	151
	M52	Bovine subclinical mastitis^1^	t529	04–34	504
	M330	Bovine subclinical mastitis^1^	t529	04–34	151
479	M126	Bovine subclinical mastitis^1^	t543	04-20-17	479

* MRSA; ^1^isolates collected in Switzerland.

### Microarray Manufacturing and Design

To compare the genetic content of the investigated micro-organisms we designed a microarray experiment based on nine fully sequenced *S. aureus* genomes. The microarray chip was manufactured by *in situ* synthesis of a set of 15,600 60-mer long oligonucleotide probes (Agilent, Palo Alto, CA, USA), selected as previously described [27]. This set of 8,877 probes covers approximately 96% of all ORFs annotated in strains USA300 [24], COL [25], RF122 [11], N315 and Mu50 [28], MW2 [29], NCTC8325 [30], as well as MRSA252 and MSSA476 [31]. Each gene was covered by one to 12 probes depending on gene length.

### Preparation of Labeled Nucleic Acids for Microarrays Probing

Purified genomic DNAs from the reference sequenced strains used for the design of the microarray chip was labeled with Cy-5 dCTP [27] and used in microarray normalization [32]. Mixtures of Cy5-labeled pooled DNAs and Cy3-labeled DNA of the test strains [33] were hybridized and scanned as previously described [34].

### Microarray Data Analysis

Hybridization fluorescence intensities were quantified using the Feature Extraction Software v9.5 (Agilent Technologies, Santa Clara, CA, USA). Local background-subtracted signals were corrected for unequal dye incorporation or unequal load of the labeled product, using a rank consistency filter and a curve-fitting algorithm per the default LOWESS (locally weighted linear regression) method. Data were analyzed using GeneSpring 8.0 (Silicon Genetics, Redwood City, CA, USA) as previously described [34] and lists of probes over-represented either in human or cow strains were further investigated manually using an Excel spreadsheet. For this manual step, genomes of *S. aureus* strains showing a hybridization signal value ≥ to 50% of the lowest value obtained with the genome of a reference strain, known to carry the corresponding gene, were considered as carrying a corresponding gene homolog. This 50% threshold was validated by PCR amplification of several genes (data not shown). The complete microarray dataset (accession number GPL7137) is posted on the Gene Expression Omnibus database (http://www.ncbi.nlm.nih.gov/geo/).

### Multiplex PCR for the Amplification of the *int* Genes of β-hemolysin Converting Prophages and of their Insertion-target Gene (*hlb*) in the *S. aureus* Chromosome

We further assessed the presence or absence of *int* genes of β-hemolysin converting prophages and their chromosomal insertion-target gene *hlb* genes by PCR. The multiplex PCR reaction mixture was as follows: 250 µg of *S. aureus* genomic DNA, MgCl_2_ 0.5 mM, dNTPs 0.2 mM, each of the following primers 0.2 µM (hlb-2∶5′-AGCTTCAAACTTAAATGTCA-3′; hlb-527∶5′-CCGAGTACAGGTGTTTGGTA-3′; ΦN315int-for: 5′-GCTTTGAAATCAGCCTGTAG-3′), GoTaq® 2.5 U in 25 µL 1X white buffer. PCR reactions were performed in a T Professional PCR thermocycler (Biometra, Goettingen, Germany). GoTaq®, white buffer, and dNTPs were from Promega (Madison, WI, USA). Primers were purchased from Microsynth AG (Balgach, Switzerland) and were described previously [19]. All other chemicals were from Sigma-Aldrich (Saint Louis, MO, USA).

### Genome Sequencing and Assembly

Total genomic DNA was isolated from the bovine *S. aureus* strain M186 using a protocol adapted from reference [35]. Bacterial cells from an overnight culture in Tryptic Soy Broth (TSB) were pelleted and resuspended in Tris-EDTA (10 mM Tris-Cl, 1 mM EDTA; pH 7.5) containing 400 µg/mL of lysostaphin (Sigma-Aldrich). After 45 min incubation at 37°C, six volumes of Nuclei lysis solution (Promega) were added and the mixture was transferred to 80°C for 10 min. After cooling the sample to room temperature, 50 µg/mL RNAse A (Sigma-Aldrich) were added and a new incubation step of 30 min. at 37°C was performed. 1/3.5 (vol/vol) of protein precipitation solution (Promega) was added and sample was left on ice for 5 min, before it was centrifuged for 10 min at 4°C. The supernatant was transferred to 1 volume isopropanol, thoroughly mixed and centrifuged at 4°C for 10 min. The DNA pellet was washed with 1 volume ethanol 70% and resuspended with 20 µL ultrapure H_2_O. In order to solubilize the genomic DNA, overnight incubation at 4°C and further at 65°C for 1 h were performed. The genomic DNA was finally stored at −20°C. Genome sequencing was performed with a Genome Analyzer IIx (Illumina Inc., San Diego, CA, USA) at the Genomic Technologies Facility of the University of Lausanne. A paired-end library with approximately 600 bp insert was constructed from 5 µg of genomic DNA and 28 million paired-end 36 bp reads were obtained following manufacturer’s instructions. In these conditions, the theoretical coverage based on the average of published genome size for *S. aureus* (ca. 2.8×10^6^ bp) was 720×. The quality of the data obtained from the sequencing was verified using FastQC (http://www.bioinformatics.bbsrc.ac.uk/projects/fastqc/). Since most of the reads were of excellent quality (data not shown), no trimming was required. Reads of insufficient quality or contaminant sequences (less than 1%) were removed using locally developed scripts (available upon request). The assembly was performed using first SOAPdenovo [36], with kmers ranging from 19 to 35, and Gapcloser (http://soap.genomics.org.cn/about.html#resource2). ORFs were detected using ORF finder and potential functions were assigned using blastp and blastn (softwares available on the National Center for Biotechnology Information (NCBI) website (http://www.ncbi.nlm.nih.gov/)).

### Minimum Inhibitory Concentrations (MICs) of Sodium Arsenite

The MICs of sodium arsenite were determined in TSB for *S. aureus* isolates carrying or not the new SCC element, using a standard broth macro-dilution method [37]. The MIC was defined as the lowest concentration of sodium arsenite that inhibited visible bacterial growth following incubation for 24 h at 37°C. A minimum of three independent experiments were performed. Sodium arsenite (NaAsO2) solution was purchased from Sigma-Aldrich.

## Results

### Clustering of Strains According to the Presence or Absence of USA300-specific Genes

**Figure 1:**
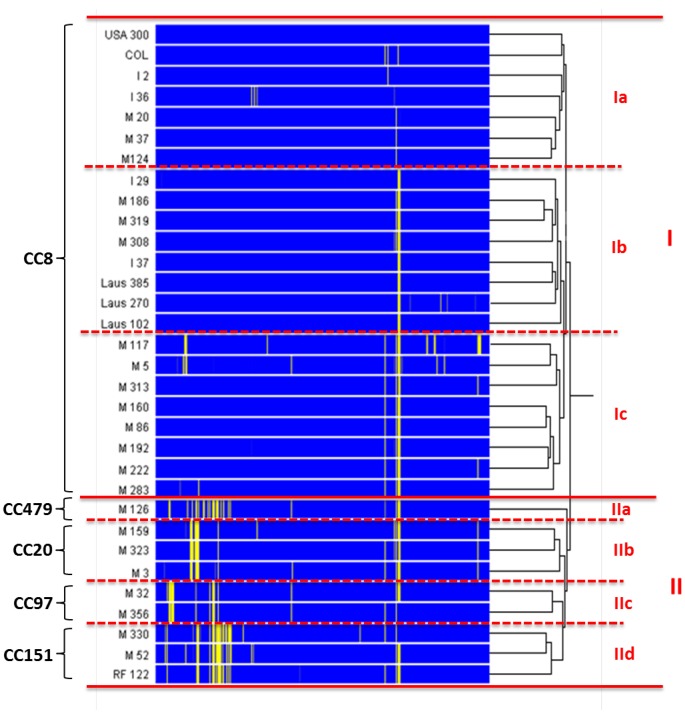
Clustering analysis, using Spearman correlation, of patterns of genome hybridization to probes matching 2,609 genes carried by the chromosome of strain USA300. Each probe set (i.e. collection of all probes hybridizing to USA300 genes) is represented by a single row of colored boxes. The blue areas correspond to genes showing significant fluorescent signal (i.e present in a corresponding genome), whereas yellow bars indicate genes poorly or not fluorescent (i.e. absent from a corresponding genome). The dendrogram on the right of the figure (black lines) represents the similarity matrix of the strain set. Clonal clusters (CCs) are indicated on the left. Clusters and sub-clusters are indicated by roman letters on the right.

To evaluate the relatedness between the various isolates, the genomes of the 32 tested organisms (Table 1) were evaluated for the presence or absence of 2,609 genes carried by USA300, and the obtained patterns were clustered by Spearman correlation (Figure 1). Clusters and sub-clusters were very similar to those recently reported for the same isolates by amplified fragment-length polymorphism (AFLP) and multi-locus sequence typing (MLST) [17]. Two major clusters were delineated; the first called cluster I, regrouped only CC8 strains, and the second called cluster II, contained all the non-CC8 isolates. Cluster I further segregated in three sub-clusters, among which sub-clusters Ia and Ib consisted of a mix of human and bovine CC8 strains that were relatively close to USA300, and sub-cluster Ic contained only CC8 isolates of bovine origin. Cluster II contained only bovine strains, but segregated in sub-clusters as well (Figure 1). Indeed, CC479, CC20, CC97, and CC151 isolates regrouped separately into four sub-clusters, named IIa, IIb, IIc, and IId, respectively (Figure 1). Thus, while clusters I and II broadly segregated between rather human types and typical bovine types of isolates, sub-clustering within CC8 strains further delineated differences between human and bovine CC8 isolates.

### Comparing Human and Bovine CC8 Isolates by Microarray

1,816 genes were found to be present on the genomes of all tested CC8 strains and corresponded to the so-called CC8 core genome (data not shown). Amongst the 8,877 60-mer DNA probes represented on the microarray chips, 198 (2.2%), corresponding to 127 genes, were found to have a higher prevalence in human than in bovine CC8 isolates. Moreover, out of these 127 genes, 95 (74.8%) were related to bacteriophage genes, 19 (15%) to *S. aureus* pathogenicity islands (SaPIs) genes, and 11 (8.7%) to staphylococcal cassette chromosome *mec* (SCC*mec*) genes. Thus, >99% of the genes associated with human specificity were carried by MGEs.

In symmetry, 43 probes (0.48%) corresponding to 29 genes, were over-represented in bovine CC8 isolates. Out of these 29 genes, 14 (48.3%) were homolog to genes carried by diverse SCC*mec* elements, eight (27.6%) corresponded to genes carried by the *S. aureus* pathogenicity island 5 (SaPI5), and seven (24.1%) were related to transposon genes. Thus, all genes associated with bovine specificity were also carried by MGEs.

Altogether, the human and bovine CC8 genomes differed only by a total of 156 genes, of which 154 (98.7%) were carried by MGEs. Below, we attempted to sort out which of these genes, or set of genes, might be the most likely candidates to promote specificity of *S. aureus* CC8 strains for either the human or the bovine host.

### Comparing MGEs Gene Content

Since close to 99% of the genetic differences between human and bovine CC8 isolates were related to MGEs, we concentrated on these elements for further analyses. More precisely, we evaluated our whole strain collection for the presence or absence of homolog genes to every single gene carried by the major MGEs found in the two human CC8 reference strains USA300 and COL, as well as the bovine CC151 reference strain RF122.

### With Respect to the Genomic Islands vSaα and vSaβ

The non-phage and non-SCC vSaα and vSaβ genomic islands are well conserved in all sequenced *S. aureus* [38]. Therefore, they were expected to be present in most of the studied isolates. Accordingly, both CC8 and typical bovine clusters uniformly carried several genes that were homolog to those of the vSaα and vSaβ of USA300 (Table S1 and S2, respectively). Nevertheless, while the entire group of CC8 strains presented quite uniform patterns for both vSaα and vSaβ, they were clearly different from the patterns found in typical bovine clusters, in which even inter-cluster differences were observed. Thus, the CC8 strains were clearly different from the typical bovine clusters in this respect. Moreover, this segregation was further confirmed when the strain collection was compared to the vSaα and vSaβ of COL and the reference bovine strain RF122 (Table S8–S9 and S10–S11, respectively).

### With Respect to the USA300 Prophages ΦSa2 and ΦSa3

USA300 is lysogenized by two bacteriophages. ΦSa2 carries the Panton-Valentine Leukocidin (PVL) [39], and ΦSa3, which is a member of a family of β-hemolysin converting bacteriophages that share a very similar integrase *int* (genes). Of note, ΦSa3 and related prophages may harbor determinants implicated in immune evasion [40], including a staphylokinase (SAK), a chemotaxis inhibitory protein (CHIPS), and the staphylococcal complement inhibitor SCIN.

Homologs of USA300 ΦSa2 prophage, devoid of the PVL *lukF-PV* and *lukS-PV* genes, were only found in the CC8 sub-cluster Ia, which contained USA300 and a few human and bovine CC8 strains, as well as in two typical bovine strains of sub-clusters IIc and IId (Table S3). Thus, ΦSa2 did not discriminate between human and bovine isolates.

In sharp contrast, ΦSa3-related β-hemolysin converting prophages, were present in the two mixed human-bovine CC8 sub-clusters Ia and Ib (except for COL), but notoriously absent from the bovine-only CC8 sub-cluster Ic, as well as from all the typical bovine clusters (Table S4). This observation was in agreement with the fact that such prophages are typically associated with human *S. aureus* isolates, but tend to be absent from animal strains [11], [20], [23]. Thus, the presence or absence of β-hemolysin converting prophages made a further distinction between sub-clusters Ia and Ib, which contained mixed human-bovine CC8 isolates, and sub-cluster Ic that contained bovine-only CC8 isolates. Indeed, strains of the sub-cluster Ic, lacking β-hemolysin converting prophages, were closer to typical bovine strains in this regard. To further determine the chromosomal insertion site of β-hemolysin converting prophages, we performed multiplex PCR reactions on genomic DNA from all strains using specific primers for the β-hemolysin converting prophage ΦN315 *int* gene and the *S. aureus* β-hemolysin (*hlb*) gene [19]. The presence of amplicons of the expected size confirmed the presence of ΦN315 *int* homologs in the genomes of the isolates harboring β-hemolysin converting prophages (not shown). Moreover, no amplification was obtained for the chromosomal *hlb* gene, supporting the fact that this gene was interrupted by the integration of the prophage, as described elsewhere [19]. Of note, while all the identified β-hemolysin converting prophages carried homologs to the typical ΦSa3 *sak* and *scin* genes, only 6/18 of them carried homologs to the ΦSa3 *chips* gene.

### With Respect to Other Non-SCC MGEs

Other non-SCC MGEs examined herein included the USA300 SaPI5 (Table S5); a USA300 transposon-related region (Table S6); the COL prophage ΦSaCOL, which is closely related to ΦSa2 (Table S12); the COL SaPI3 (Table S13); as well as the bovine RF122 SaPIbov [41], SaPIbov3 [42], vSabov, and prophage ΦRF122 (Table S14, S15, S16, and S17 respectively). None of these elements were discriminatory between human and animal isolates except for the bovine genomic island SaPIbov3, which was only present in typical bovine clusters but not in CC8 strains. This further supported the fact that bovine CC8 strains were more closely related to human CC8 than to typical bovine strains (Table S15).

### With Respect to the USA300 SCC*mec* Cassette

SCC*mec* is a genomic island conferring methicillin resistance [43]. It is found in MRSA USA300, but not systematically in other *S. aureus* isolates. Table S7 shows that only two strains (i.e. MRSA I2 and COL) contained relatively numerous gene homologs, including *mecA/mecRI*, to the USA300 SCC*mec,* which was consistent with the fact that they were MRSA. Strikingly, a different and restricted stretch of gene homologs was uniquely present in all bovine CC8 isolates, but never found in human CC8 strains or isolates of the typical bovine clusters. This region appeared as a truncated SCC, which carried homologs of the *ccrA* and *ccrB* recombinase genes, as well as a few other determinants present on the SCC*mec* of USA300. However, it lacked the methicillin resistance determinants *mecA*/*mecR1* and surrounding gene (i.e. from *sausa300_0027* to sausa300_*0035*) (Table S7). This *mecA*-negative SCC element discriminated the bovine CC8 strains from all other strains of the present collection, be it CC8 or typical bovine CCs, and this observation was confirmed by comparison with COL SCC*mec* (Table S18).

### Genetic Organization of the Representative Non-*mecA* SCC Cassette from Bovine CC8 Strain M186 (SCC^M186^)

**Figure 2:**
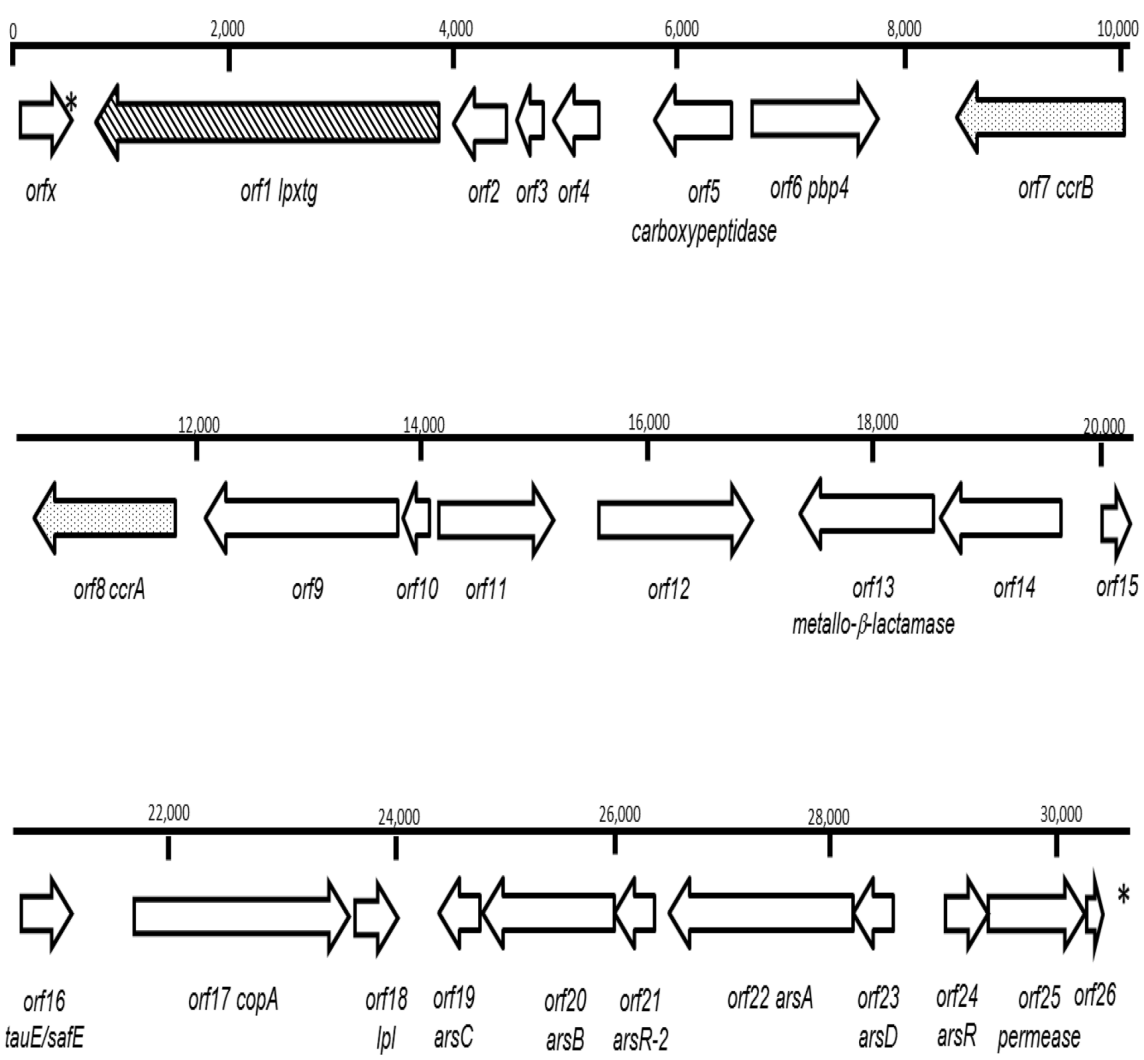
Schematic map of SCC^M186^. Genes are represented by black arrows pointing in the direction of transcription. The positions of *att*L and *att*R flanking the cassette are indicated by asterisks. The gene coding for the potential new LPXTG (*orf1*) is represented by an oblique dashed arrow. *CcrB* (*orf7*) and *ccrA* (*orf8*) are represented by dotted arrows.

Bovine CC8 strains were specifically associated with the presence of a truncated SSC cassette, which was devoid of the *mecA* gene. Thus, the nucleotide sequence of this cassette was further extracted and annotated from the preliminary draft chromosomal sequence of strain M186, and named SCC^M186^. After assembly of the reads generated by Illumina with SOAPdenovo and GapCloser, we obtained 129 contigs ranging from 1,000 to 674,164 bp in length. To map SCC^M186^, we sought for the *orfx* gene, which precedes the insertion site upstream of SCC cassettes (28). *orfx* was localized on a single contig of 277,076 bp in length. A ca. 40,000 bp fragment, starting with the first nucleotide of *orfx* (i.e. designed as position one), was extracted from this contig, in which we further localized the chromosomal 15 bp direct repeats *attL* and *attR* that typically flank SCC cassettes [44]. These were found at nucleotide positions 462–476 (AGAAGCTTATCATAA) and 30,741–30,755 (AGAGGCGTATCATAA). Thus, the deduced length of SCC^M186^ was 30,279 bp and contained 26 potential ORFs (Figure 2 and Table 2). Based on its ORF sequences, SCC^M186^ appeared as a composite cassette formed by three distinct regions. From the 5′ to 3′ ends, the first region was composed of six ORFs, of which one (*orf1*) encoded for a potential new LPXTG-protein harboring a LPDTG signature, which is described below. The five other ORFs, encoded by *orf2* to *orf6*, showed high degrees of amino acids identity (i.e. from 86 to 98%) with ORFs regrouped on a unique region encompassing SE0030 to SE0035 on the genome of *S. epidermidis* strain ATCC12224 (Table 2). *orf2*, *orf3* and *orf4* coded for three hypothetical proteins which were also found in USA300 (SAUSA300_0056, 0057, and 0059, respectively). *Orf5* encoded for a carboxypeptidase and *orf6* for a putative penicillin-binding protein 4.

**Table 2:** Genetic composition of SCC^M186^.

ORF number on SCC^M186^	Homolog to	% of amino acids identity	Gene product
1	HMPREF9994_12940	74	Hypothetical protein
	(last 400 aa)		
	GT20_0444	95	
	(PEG/QPGN domain)		
2	SE0030	86	Hypothetical protein
	SAUSA300_0056	88	
3	SE0031	89	Hypothetical protein
	SAUSA300_0057	91	
4	SE0033	91	Hypothetical protein
	SAUSA300_0059	90	
5	SE0034	98	Carboxypeptidase
6	SE0035	94	PBP4
7	SE0036	92	CcrB
	SAUSA300_0037	92	
8	SE0037	96	CcrA
	SAUSA300_0038	90	
9	SE0038	84	Hypothetical protein
	SAUSA300_0039	99	
10	SAUSA300_0040	100	Hypothetical protein
11	SAUSA300_0041	96	Hypothetical protein
12	SAUSA300_0042	99	Transcriptional regulator
13	SE0129	98	Metallo-β-lactamase
	SAUSA300_0044	95	
14	SE0130	93	Rhodanese domain protein
15	SE0132	99	Hypothetical protein
16	SE0133	93	Sulfite exporter TauE/SafE
17	SE0126	97	CopA
	SAUSA300_0078	97	
18	SE0128	97	Putative lipoprotein
	SAUSA300_0079	91	
19	SE0134	92	ArsC
	SAUSA300_1719	80	
20	SE0135	92	ArsB
	SAUSA300_1718	80	
21	SE0136	100	ArsR
	SAUSA300_1717	58	
22	SE0137	99	ArsA
23	SE0138	99	ArsD
24	SE0139	100	ArsR
25	SE0140	99	Putative permease
26	SE0141	100	Hypothetical protein

Best hits obtained with blastp against the non-redundant protein database are shown. SExxxx and SAUSA_xxxx represent ORFs found in *S. epidermidis* strain ATCC 12228 and *S. aureus* strain USA300_FPR3757, respectively. HMPREF9994_12940 and GT20_0444 are found in *S. epidermidis* strain NIHLM088 and *G. thermoglucosidasius* strain TNO-09.020.

The central region was composed of six genes showing a conserved organization with the *sausa300_0037* to *_0042* genes of the USA300 SCC*mec*. Within this region, *orf8* and *orf7* encoded for the recombinases CcrA and CcrB, respectively. The *ccrA* and *ccrB* genes were members of the *ccr* allotype II and both proteins showed 90 and 92% identity, respectively, at the amino acid level with corresponding proteins in USA300. The gene products of *orf9*, *orf10*, and *orf11* were annotated as hypothetical proteins with very high (i.e. ≥96%) amino acid identity to USA300 proteins SAUSA300_0039, 0040, and 0041, respectively. Eventually, ORF12 of SCC^M186^, showed 99% identity to USA300_0042, which could act as a transcriptional regulator.

The third region, starting with *orf13*, corresponded to a region spanning from *se0129* to *se0141* in *S. epidermidis* ATCC 12224 with only slight gene shuffling (*se0126* and *se0128*) and two gene deletions (*se0127* and *se0131*). This region carried several resistance determinants (see Table 2 for homologies at the amino acid level), including a metallo-β-lactamase (*orf13*), a putative cyanide-resistance gene (*orf14*) [45], a sulfite exporter (*orf16*), a copper-resistance gene (*orf17*) [46], and an arsenic-resistance operon (*orf19* to *orf24* corresponding to *se0134 to se0139*). This region also carried a lipoprotein gene (*orf18)*, which could be involved in virulence [47], [48], [49].

Since resistance to chemicals such as arsenic may be pertinent in the agricultural environment, we tested the susceptibility to sodium arsenite of bovine CC8 isolates, carrying the new SCC cassette, as compared to all other strains of the collection, which did not carry the new SCC. The MIC of arsenite was 25 mM for all the bovine CC8 isolates, including M186. In contrast, it ranged between 0.4 to 3 mM in all other strains, i.e. up to 8 times lower than in SCC-positive strains.

### New SCC^M186^-related LPXTG Protein

The deduced amino acid sequence of the *orf1*-encoded LPXTG-protein of SCC^M186^ was composed of 1,151 amino acids and had a theoretical molecular weight of ca. 124 kDa and a pI of 4.47 using Compute pI/Mw tool (http://web.expasy.org/compute_pi/). A search for conserved domains [50] identified an YSIRK type signal peptide (YSIRKxxxGxxSIA, pfam04650) at position 23–35 and two G5 domains (pfam07501). Interestingly, the LPXTG-protein harbored by SCC^M186^ showed significant homologies (74% over the 400 amino acids at Cterminal) to SE0175, a putative accumulation associated protein (AAP) found in *S. epidermidis* ATCC 12224. Moreover, an LPDTG signature of *S. aureus* adhesins was manually found at position 1112–1116. Finally, the LPDTG motif was preceded by 25 proline-rich PE/GQPGN repeats, which showed 95% of homology with a domain harbored by a potential surface LPXTG-protein (GT20_0444) of hypothetical function found in the environmental bacterium *G. thermoglucosidasius* TNO-09.020.

## Discussion

The present results indicate a clear segregation between *S. aureus* strains from the CC8 cluster and typical bovine CCs. In addition, they also show that some isolates of the supposedly human-only CC8 cluster had permeated the bovine environment, as bovine CC8 isolates resembled much more isolates of the human CC8 than isolates of the typical bovine clusters. These observations may provide clues for the speculated jump of CC8 strains from human to cattle [17].

Having assessed that 99% of the genetic differences observed between the tested isolates resided in MGEs, we found that several of them were not discriminative at all, because they were not systematically represented in particular clusters. On the other hand, a group of MGEs appeared to be present in all strains, but demonstrated discrete differences in gene contents between CC8 isolates and isolates from typical bovine CCs. These included the genomic islands vSaα and vSaβ, which are believed to have evolved with *S. aureus* since a long time, and are present in all the strains sequenced so far [38]. In our study, both islands adopted clear patterns that differentiated the CC8 group (including human and bovine strains) from typical bovine CCs. This suggested that both vSaα and vSaβ emerged from a common ancestor and further evolved divergently in either the human or the bovine environment. Hence, the fact that bovine CC8 isolates shared very similar vSaα and vSaβ with human CC8 isolates, supported the hypothesis that they were originally human, and had jumped into cattle at a more recent occurrence in time. Moreover, this hypothesis was further supported by the fact that typical SaPIbov3 homologs were strikingly absent in CC8 isolates. Indeed, typical genes of this island were recently reported to discriminate *S. aureus* isolated in cattle with mastitis from human clinical strains [42].

Additional MGEs helped determine even more specific differences within the human and bovine CC8 isolates. These were exemplified by β-hemolysin converting prophages and a new composite SCC cassette. β-hemolysin converting prophages were present in the two CC8 sub-clusters Ia and Ib, which contained a mixture of human and bovine strains, but were absent from the bovine-only CC8 sub-cluster Ic, as well as from all the typical bovine CCs. This was highly reminiscent of recent studies on *S. aureus* jumps between human and small ruminant, poultry, and pig [15], [22], [23]. In all cases the postulated human to animal jump was associated with the loss of β-hemolysin converting prophages from the human strains, along with their establishment in animals. Since such prophages disrupted the *S. aureus* chromosomal *hlb* gene, encoding for hemolysin β it was proposed that this toxin was either unnecessary for persistence of *S. aureus* in humans, or even detrimental for it. On the other hand, it could be advantageous in animals [19]. Accordingly, the finding that human-derived CC8 isolates lose β-hemolysin converting prophages upon transition to becoming bovine-adapted is a strong evidence of a significant role of β-hemolysin in the process of host adaptation in cows.

Likewise, adaptation of *S. aureus* to a new host is frequently associated with the acquisition of new genetic determinants such as pathogenicity islands, additional prophage(s), or new SCC islands [15], [22], [23]. In the present observation, the bovine CC8 isolates have acquired additional features that possibly helped them settle in their new environment. This was substantiated by the the new *mec*-negative SCC which was present only in bovine CC8 isolates, but never in human CC8 or typical bovine CCs. This SCC was reminiscent of the SCC*mec* acquired by porcine *S. aureus* CC398 [15], [22], [23], as it also carried genes conferring resistance to toxic agents (e.g. arsenic and copper). In the strains described herein, the MIC of sodium arsenite was uniformly 25 mM for all strains harboring the new SCC element, as compared to ≤3 mM for all strains that were devoid of it. This observation indicates that all SCC+ strains carried a SCC equipped with a functional arsenic-resistance operon that could represent an asset for survival in the agricultural milieu. Although the new SCC shared the same *ccrAB* allotype II with the SCC*mec* of USA300 and *S. epidermidis* ATCC 12228, it was a composite element composed of homologs to regions found in *S. aureus*, *S. epidermidis* and environmental bacteria. This chimeric construction indicates that it was not just the descendant of an existing human SCC*mec* parent, but rather *de novo* (re)-constructed from parts of different genomes, most likely in the rural environment.

Of highest interest, was the fact that it carried a gene encoding for a new LPXTG protein of unknown function, which was partly homologous to a protein found in the environmental bacterium *G. thermoglucosidasius* TNO-09.020 [51]. The presence of this LPXTG-protein may well be explained by horizontal gene transfer from a Geobacillus sp., a genus known as potential milk contaminant [52]. *S. aureus* LPXTG proteins are involved in various functions, including host colonization in which they play crucial roles in bacterial adhesion to host tissues, and are therefore termed adhesins [53]. The presence of a signal peptide which is found in many staphylococcal surface proteins, and two G5 domains to which a N-acetylglucosamine binding function has been attributed [54], strongly suggests an adhesin function for this protein. This possibility is reinforced by the significant homology with a *S. epidermidis* AAP. Indeed, such proteins have been shown to play major roles in the accumulation of *S. epidermidis* on polymer surfaces, and thus biofilm formation [55], [56].

**Figure 3:**
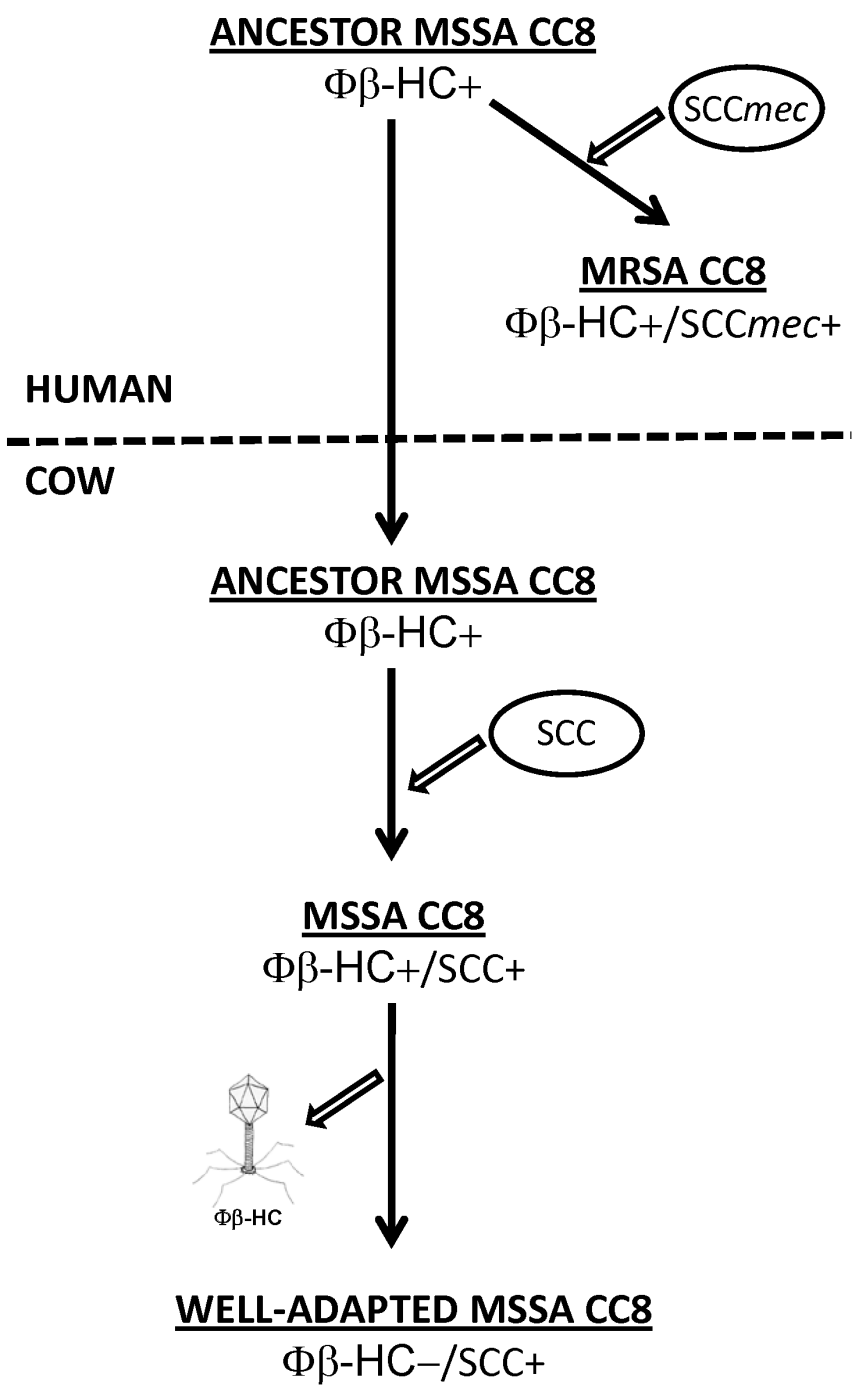
Schematic representation of the proposed scenario for the human to cow jump of *S. aureus* CC8 strains. The human CC8 MSSA ancestor strain carried a β-hemolysin converting prophage (Φβ-HC), which is suggested to be important for survival in the human environment. The upper part of the Figure shows the acquisition of SCC*mec* by such ancestor, leading to human MRSA such as USA300. The lower part of the Figure shows the progressive passage to the bovine environment, which includes first the acquisition of the new *mecA*-negative SCC, and then the loss of the β-hemolysin converting prophage.

Taken together, the present work is an additional illustration of the adaptability of *S. aureus* to various hosts and the subtlety of the biological tools underlying it. We obtained convincing evidences supporting the human to bovine jump scenario of *S. aureus* CC8 rather than the contrary. We therefore propose that bovine CC8 strains originated from human CC8 strains following a scenario depicted in Figure 3. This raises several academic and public health issues. One is the contribution of the new SCC to bovine colonization and/or infection, and whether it may definitively hold the bovine CC8 strain in the bovine milieu. Another is whether this new island could acquire a *mec*A*/mec*RI complex and further spread methicillin resistance both in cattles and humans. Such a precedent recently occurred in the swine-related MRSA CC398, which first jumped from human to pig and then jumped back equipped with SCC*mec*. In view of this case, bovine CC8 strains might well be a new threat for human and veterinary medicine, which deserve concern and preventive control.

## Supporting Information

Table S1 **Distribution of USA300 vSaα island homolog genes on the genomes of the tested strains.** Black rectangles represent genes that are present on corresponding genomes.
(TIFF)

Table S2 **Distribution of USA300 vSaβ island homolog genes on the genomes of the tested strains.** Black rectangles represent genes that are present on corresponding genomes.
(TIFF)

Table S3 **Distribution of USA300 prophage ΦSa2 homolog genes on the genomes of the tested strains.** Black rectangles represent genes that are present on corresponding genomes.
(TIFF)

Table S4 **Distribution of USA300 prophage ΦSa3 homolog genes on the genomes of the tested strains.** Black rectangles represent genes that are present on corresponding genomes.
(TIFF)

Table S5 **Distribution of USA300 pathogenicity island SaPI5 homolog genes on the genomes of the tested strains.** Black rectangles represent genes that are present on corresponding genomes.
(TIFF)

Table S6 **Distribution of homologs of genes found on a USA300 transposon-related region on the genomes of the tested strains.** Black rectangles represent genes that are present on corresponding genomes.
(TIFF)

Table S7 **Distribution of USA300 SCC** ***mec*** **homolog genes on the genomes of the tested strains.** Black rectangles represent genes that are present on corresponding genomes.
(TIFF)

Table S8 **Distribution of COL vSaα island homolog genes on the genomes of the tested strains.** Black rectangles represent genes that are present on corresponding genomes.
(TIFF)

Table S9 **Distribution of COL vSaβ island homolog genes on the genomes of the tested strains.** Black rectangles represent genes that are present on corresponding genomes.
(TIFF)

Table S10 **Distribution of RF122 vSaα island homolog genes on the genomes of the tested strains.** Black rectangles represent genes that are present on corresponding genomes.
(TIFF)

Table S11 **Distribution of RF122 vSaβ island homolog genes on the genomes of the tested strains.** Black rectangles represent genes that are present on corresponding genomes.
(TIFF)

Table S12 **Distribution of prophage ΦSaCOL homolog genes on the genomes of the tested strains.** Black rectangles represent genes that are present on corresponding genomes.
(TIFF)

Table S13 **Distribution of COL pathogenicity island SaPI3 homolog genes on the genomes of the tested strains.** Black rectangles represent genes that are present on corresponding genomes.
(TIFF)

Table S14 **Distribution of RF122 pathogenicity island SaPIbov homolog genes on the genomes of the tested strains.** Black rectangles represent genes that are present on corresponding genomes.
(TIFF)

Table S15 **Distribution of RF122 pathogenicity island SaPIbov3 homolog genes on the genomes of the tested strains.** Black rectangles represent genes that are present on corresponding genomes.
(TIFF)

Table S16 **Distribution of RF122 vSabov island homolog genes on the genomes of the tested strains.** Black rectangles represent genes that are present on corresponding genomes.
(TIFF)

Table S17 **Distribution of prophage ΦRF122 homolog genes on the genomes of the tested strains.** Black rectangles represent genes that are present on corresponding genomes.
(TIFF)

Table S18 **Distribution of COL SCC** ***mec*** **cassette homolog genes on the genomes of the tested strains.** Black rectangles represent genes that are present on corresponding genomes.
(TIFF)
